# Development of fluorescent *Plasmodium falciparum *for *in vitro *growth inhibition assays

**DOI:** 10.1186/1475-2875-9-152

**Published:** 2010-06-03

**Authors:** Danny W Wilson, Brendan S Crabb, James G Beeson

**Affiliations:** 1 Infection and Immunity Division, the Walter and Eliza Hall Institute of Medical Research, 1G Royal Parade, Parkville, Victoria, 3050, Australia; 2 Department of Medical Biology, University of Melbourne, Victoria, Australia; 3 Macfarlane Burnet Institute for Medical Research and Public Health, Melbourne, Victoria 3004, Australia

## Abstract

**Background:**

*Plasmodium falciparum in vitro *growth inhibition assays are widely used to evaluate and quantify the functional activity of acquired and vaccine-induced antibodies and the anti-malarial activity of known drugs and novel compounds. However, several constraints have limited the use of these assays in large-scale population studies, vaccine trials and compound screening for drug discovery and development.

**Methods:**

The D10 *P. falciparum *line was transfected to express green fluorescent protein (GFP). *In vitro *growth inhibition assays were performed over one or two cycles of *P. falciparum *asexual replication using inhibitory polyclonal antibodies raised in rabbits, an inhibitory monoclonal antibody, human serum samples, and anti-malarials. Parasitaemia was evaluated by microscopy and flow cytometry.

**Results:**

Transfected parasites expressed GFP throughout all asexual stages and were clearly detectable by flow cytometry and fluorescence microscopy. Measurement of parasite growth inhibition was the same when determined by detection of GFP fluorescence or staining with ethidium bromide. There was no difference in the inhibitory activity of samples when tested against the transfected parasites compared to the parental line. The level of fluorescence of GFP-expressing parasites increased throughout the course of asexual development. Among ring-stages, GFP-fluorescent parasites were readily separated from uninfected erythrocytes by flow cytometry, whereas this was less clear using ethidium bromide staining. Inhibition by serum and antibody samples was consistently higher when tested over two cycles of growth compared to one, and when using a 1 in 10 sample dilution compared to 1 in 20, but there was no difference detected when using a different starting parasitaemia to set-up growth assays. Flow cytometry based measurements of parasitaemia proved more reproducible than microscopy counts.

**Conclusions:**

Flow cytometry based assays using GFP-fluorescent parasites proved sensitive and highly reproducible for quantifying the growth-inhibitory activity of antibodies and anti-malarials, with superior reproducibility to light microscopy, and are suitable for high-throughput applications.

## Background

Malaria is a leading cause of morbidity and mortality globally and the majority of malaria-related mortality is caused by *Plasmodium falciparum*, particularly among children [1-3]. Antibodies are thought to be an important component of naturally acquired protective immunity against *P. falciparum *malaria. A number of studies suggest an association between antibody levels to merozoite antigens measured by enzyme linked immunosorbent assays (ELISA) and protection from malaria disease [4-15]. However, ELISA results are affected by the purity, folding, and boundaries of the recombinant proteins used and cannot distinguish between functional antibodies that may contribute to protection and non-functional antibodies or blocking antibodies that could potentially interfere with the activity of protective antibodies in human samples [16-20].

Studies using rabbit polyclonal sera and monoclonal antibodies (MAb) raised by vaccination with recombinant merozoite antigens or affinity purified human antibodies suggest that antibodies against merozoite antigens are capable of interfering with merozoite invasion of the RBC directly in *in vitro *assays [21-27]. *Plasmodium falciparum *asexual stage growth inhibition assays have been used extensively in malaria research to measure the levels of growth inhibitory antibodies in clinical and pre-clinical studies [16,28-35]. Growth inhibition assays are also used extensively for testing the sensitivity of isolates to anti-malarial drugs or screening compounds for anti-malarial activity in drug development studies.

The most readily available, simplest and most widely used method for measuring parasitaemia in growth inhibition assays is light microscopy [29,30,33,36-40]. However, microscopy of thin films is labour-intensive, with studies suggesting that the reproducibility of parasitaemia counts decreases with decreasing parasite density and is affected by the distribution of parasites on the slide [41,42].

High throughput approaches such as parasite lactate dehydrogenase (pLDH), and amino acid incorporation assays (hypoxanthine and isoleucine uptake assays) allow easier assessment of parasite growth for large sample sets [17,31,32,34,35,39,43,44]. However, since methods such as pLDH, hypoxanthine and isoleucine uptake assays measure metabolic activity, reduced signal may be caused by inhibition of intraerythrocytic parasite development as well as inhibition of invasion [43,45]. Flow cytometry-based methods have been shown to be highly reproducible, scalable for efficient use in large sample sets and comparable in terms of sensitivity to alternative methods such as microscopy, hypoxanthine uptake and pLDH assays [28,43,45-48].

In this study, growth inhibition assays were optimized and GFP-labelled fluorescent parasites developed and evaluated in growth inhibition assays using antibodies and anti-malarials. Furthermore, the sensitivity and reproducibility of flow cytometry for measuring parasite growth inhibition by antibodies was examined and compared to light microscopy. Previously, O'Donnell *et al *developed transgenic *P. falciparum *parasites expressing an antigenically distinct 19 kDa fragment of the merozoite surface protein 1 (MSP1-19) orthologue from the rodent malaria *Plasmodium chabaudi *[39]; evaluating inhibition of the *P. chabaudi *MSP1-19 line versus parasites with the *P. falciparum *MSP1-19 sequence allowed measurement of *P. falciparum *MSP1-19-specific inhibitory antibodies. In this study, MSP1-19 specific growth inhibition assays were used as a model as it allowed antigen-specific inhibition to be distinguished from non-specific inhibitory activity. MSP1-19 appears to be a target of protective immunity and is an important vaccine candidate currently under development.

## Methods

### Continuous culture of *P. falciparum*

*Plasmodium falciparum *parasites were cultured in Petri dishes using human O^+ ^erythrocytes (RBC) maintained at 2% haematocrit [43]. Parasites were grown in RPMI-HEPES culture medium (pH 7.4) supplemented with 50 μg/ml hypoxanthine, 25 mM NaHCO_3_, 20 μg/ml gentamicin, 5% (vol/vol) heat inactivated pooled human serum (various blood groups, collected by the Australian Red Cross) and 0.25% Albumax II (Gibco, Auckland, New Zealand). In some drug inhibition experiments 0.5% Albumax was used with no human serum added. Cultures were maintained in airtight boxes at 37°C in an atmosphere of 1% O_2_, 4% CO_2 _and 95% N_2_. Parasites used in these studies include the D10 clonal line, an isogenic D10-*P. chabaudi *MSP1-19 parasite line in which the *P. chabaudi *orthologue replaces the region of *P. falciparum *msp1 encoding MSP1-19 (known as PcMEGF), and an isogenic D10-*P. falciparum *MSP1-19 parasite line generated in the same manner as PcMEGF to act as a transfection control (PfM3').

### PfMSP1-19 specific growth inhibition assays

The protocol for measurement of antibody inhibition of parasite growth used in this study was modified from Persson *et al *[43]. PfMSP1-19 and PcMSP1-19 transfected lines were maintained on 24 ng/ml pyrimethamine (to select for MSP1-19 transfectants, Sigma Aldrich, St Louis, MO, U.S.A.) with 5 μg/ml blasticidin-S-HCl (to select for fluorescent transfectants, Invitrogen, Carlsbad, CA, U.S.A.) added when culturing double transfected fluorescent lines. One to two cycles prior to assay set up, parasites were removed from drug selection. Mid to late trophozoite stage cultures (30-40 hours) were washed in fresh culture medium (37°C), pelleted by centrifugation then diluted to 0.5% to 1% parasitaemia at 1% haematocrit for a one cycle assay, or 0.1% to 0.3% parasitaemia at 1% haematocrit for a two cycle assay. Fresh culture medium and fresh RBCs from two different donors warmed to 37°C were used during assay set up.

Parasitized RBCs (25 μl to 50 μl) at the appropriate parasitaemia were added to a 96 well U-bottom plate (Falcon, Becton-Dickinson, Franklin Lakes, NJ, U.S.A.). Inhibitory sample (2.5 μl to 5 μl for a 25 μl or 50 μl assay respectively) was added to the parasitized RBCs giving a 1 in 10 dilution of sample in the assay (for a 1 in 20 dilution, half the volume of sample would be added). All samples were run in duplicate. A panel of non-immune sera from Melbourne donors or PBS were included as non-inhibitory controls. The 96 well plates were placed in a humidified air-tight gassed culture box at 37°C.

One cycle ring stage assays for microscopy and flow cytometry were cultured for approximately 24 hours until young rings were visible and no schizont stage parasites remained. Thin smears of cell pellets were methanol fixed and stained with 5% Giemsa (Merck, Darmstadt, Germany). One thousand RBCs were counted for each slide. Only early to late rings (0 to 20 hours old) were counted as parasitized RBCs, with each infected RBC counted as a single invasion event, regardless of how many parasites were in the cell.

Growth assays performed over one cycle of erythrocyte invasion were cultured for 48 hours post set up until parasites were between 30-40 hours post invasion, when they were prepared for flow cytometry analysis as appropriate. Growth assays performed over two cycles of erythrocyte invasion were supplemented with 5 μl to 10 μl (25 μl or 50 μl assay respectively) of fresh culture medium warmed to 37°C, 48 hours post set up (parasites 30-40 hours post invasion). Ninety to 96 hours post assay set up the two cycle trophozoite stage assays were prepared for flow cytometry analysis. For non-fluorescent parasite lines, RBCs were resuspended in 100 μl of PBS with 10 μg/ml ethidium bromide (EtBr, Bio-Rad, Hercules, CA U.S.A.), stained for one hour before centrifugation, removal of the supernatant and resuspension in 200 μl of PBS. GFP fluorescent parasite lines were resuspended in PBS to make a final volume of 200 μl. Parasitaemia was measured on a Becton Dickinson FACSCalibur (Franklin Lakes, NJ, U.S.A.) flow cytometer using a 488 nm laser for excitation of both GFP fluorescent (Fl-1) and EtBr stained (Fl-2) parasites. Typically, 50,000 to 80,000 RBCs were counted in each well. Samples were analysed using FlowJo software (Tree Star Inc, Ashland, OR, U.S.A.) by first gating for intact erythrocytes by side scatter and forward scatter parameters, and subsequently determining the proportion of FL1 or FL2 positive cells.

In this study, when results for both the parental PcMEGF and PfM3'lines and the GFP fluorescent PcPHG and PfPHG lines (described following) are described they will be referred to as the PcMSP1-19 and PfMSP1-19 lines, respectively. For both the PcMSP1-19 and PfMSP1-19 lines, parasite growth (G) was determined by dividing the mean parasitaemia of duplicate wells for a test sample (S_mean_) by the mean parasitaemia of two or more wells of non-inhibitory control (C_mean_) and multiplying by 100 (G = {S_mean_/C_mean_}*100).

Samples used in growth inhibition assays included rabbit polyclonal antibodies raised against recombinant *P. falciparum *or *P. chabaudi *MSP1-19 [39], an inhibitory MAb raised against 3D7 AMA1 (1F9) [49,50], the anti-malarial drugs chloroquine (Sigma Aldrich, St Louis, MO U.S.A.) and quinine (Sigma Aldrich, St Louis, MO U.S.A.), serum samples from residents of Melbourne, Australia, and archived serum samples from malaria-exposed adult residents of Papua New Guinea. Ethical consent for the use of human samples and blood products was obtained from the Walter and Eliza Hall Institute, and the Medical Research Advisory Council, PNG. All human samples were collected following informed consent. Serum samples were heat inactivated at 56°C for 45 minutes prior to use in assays.

### Construction of GFP plasmids for transfection

GFP plasmids for the transfection of the PfM3' and PcMEGF MSP1-19 transfected parent lines [39,51] were modified from plasmids using Gateway ™ recombination technology (Invitrogen, Carlsbad, CA, U.S.A.) as described by Tonkin *et al *[52]. Briefly the pHGB pENTR vector [52] was digested with the restriction enzyme Xho I and gel extracted (Qiagen, Valencia, CA, U.S.A.) *P. falciparum *Rep20 sequence [53] was inserted 3' of the *P. berghei *DHFR terminator sequence. The resulting plasmid, pHGBr, was digested with the restriction enzymes Bgl II and Pst I and the original GFP sequence, which lacked a start codon, was replaced with a GFP mutation II sequence [54] containing a start codon to produce the plasmid pHGBr(ATG). The 5'flanking region of the hsp86 promoter that controls GFP expression in this plasmid has been described previously [55].

**Figure 1:**
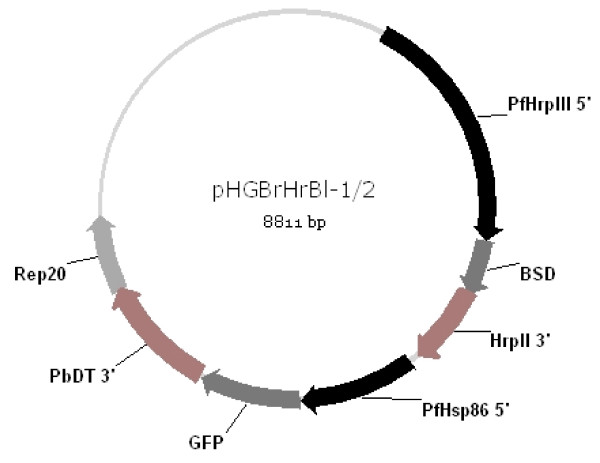
**Green fluorescent protein (GFP) transfection plasmid**. Plasmid pHGBrHrBl-1/2 was constructed and used to transfect *P. falciparum *blood-stage parasites *in vitro*. Letters and numbers forming the name of the plasmid are in bold. The plasmid has the **H**sp86 5' promoter driving **G**FP expression with a *P. berghei *3' terminator region (**B**), and **r**ep20 targeting sequence. The drug cassette contains the Pf**Hr**pIII 5' promoter driving blasticidin-S-deaminase (BSD) (**Bl**) with a PfHrpII 3' terminator. Fluorescence and drug cassettes are orientated in a head to tail orientation (5' to 3' end followed by 5' to 3' end) (**1/2**).

The pHrBl-1/2 destination vector described by Tonkin *et al *[52] containing the blasticidin-S-deaminase gene coding for resistance to blasticidin-S-HCl [56] was used as the destination vector in LR clonase reactions with the pHGBr(ATG) ENTR vector described above. The LR Clonase reactions were performed according to manufacturers instructions (Invitrogen, Carlsbad, CA, U.S.A.). The pHrBl-1/2 destination vector (2 μl) was mixed with 5 μl of the pHGBr(ATG) ENTR vector at equal concentrations (approximately 300 ng). Four microlitres of 5 × LR Clonase reaction buffer and 4 μl of LR Clonase enzyme mix were added (Invitrogen, Carlsbad, CA, U.S.A.). The reaction mixture was made up to 20 μl with TE Buffer (pH 8.0) and the reaction allowed to proceed for 1 hour at room temperature. The reaction was stopped by the addition of proteinase K (Roche Diagnostics, Mannheim, Germany) and incubation at 37°C for 10 minutes. The LR clonase reactions were then transformed directly into CcdB sensitive electrocompetent bacterial cells. The presence of the pHGBrHrBl-1/2 vector (Figure 1) in bacterial clones was confirmed by restriction digest. Plasmids for transfection were purified from 500 μl of bacterial culture using a Plasmid Maxi kit (Qiagen, Valencia, CA, U.S.A.) according to manufacturers instructions.

### Transfection of parasites with fluorescence plasmids

Transfection of *P. falciparum *parasites PfM3' and PcMEGF with the pHGBrHrBl-1/2 plasmid was undertaken as described previously [57] using modified electroporation conditions [58]. Briefly, approximately 70 μg of purified plasmid DNA was resuspended in 15 μl of warm TE buffer. Three hundred and eighty five microlitres of sterile, warm (37°C) cytomix (120 mM KCl, 0.15 mM CaCl_2_, 2 mM EGTA, 5 mM MgCl_2_, 10 mM K_2_HPO_4_/KH_2_PO_4 _pH 7.6, 25 mM HEPES pH 7.6) were added to the plasmid and then mixed with 200 μl of infected RBC with 5-8% ring stage parasites before being transferred to a 0.2 cm electroporation cuvette. The cytomix and infected RBC mix was electroporated at 950 μF capacitance and 0.31 kV (Biorad Gene Pulser II, Biorad, Hercules, CA, U.S.A.) and the infected red blood cells transferred immediately into 10 ml of culture medium containing 2% haematocrit RBCs, placed into a airtight gassed chamber and incubated at 37°C.

Drug cycling commenced two days after transfection using 5 μg/ml of blasticidin-S-HCl to select for fluorescent parasites with 24 ng/ml of pyrimethamine added to maintain integration of the MSP1-19 transfection vectors. After the second cycle of drug selection, fluorescent parasites expressing GFP were cloned by limiting dilution for use in experiments. In order to ensure maintainance of both the MSP1-19 and GFP transfection plasmids for experiments, cloned parasites were cultured with the appropriate drugs (pyrimethamine and blasticidin-S-HCl, respectively). Typically, there was no evidence for the loss of GFP fluorescence or expression of the *P. chabaudi *MSP1-19 phenotype with up to two months of continuos culture. To explore whether other isolates could be successfully transfected with the same plasmid, isolates CS2 and E8B, derived from the ITG line, were also transfected.

Successful transfection of parasites with GFP plasmids was confirmed by live parasite fluorescence microscopy using an upright Zeiss Axioskop fluorescence microscope (Carl Zeiss, Jena, Germany) with parasite nuclei stained with 0.5 μg/ml DAPI (Sigma Aldrich, St Louis, MO, U.S.A.). For flow cytometry GFP fluorescent lines were stained with EtBr and the number of fluorescent parasites were quantified using a FACSCalibur and analysed using FlowJo software.

## Results

### Inhibitory polyclonal and monoclonal antibodies

**Figure 2:**
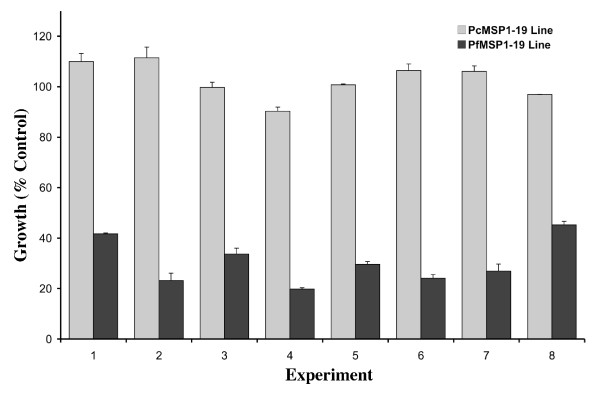
**Specificity and reproducibility of growth inhibition by PfMSP1-19 specific antibodies**. PfMSP1-19 specific inhibitory rabbit antiserum was added at a 1 in 10 dilution in two cycle growth assays. Growth is expressed as a percentage of controls (sera from non-exposed Australian residents). Results are from 8 different experiments carried out between December 2005 and July 2007. Assays were performed in duplicate and values represent the mean + range.

Polyclonal rabbit antibodies raised against PfMSP1-19 and PcMSP1-19 and a MAb raised against 3D7 AMA1 [49,50] were tested against PfMSP1-19 and PcMSP1-19 lines for growth-inhibitory activity; the PcMSP1-19 line contains the orthologous *P. chabaudi *MSP1-19 sequence in place of the endogenous *P. falciparum *sequence [39]. The PfMSP1-19 rabbit antiserum was found to specifically inhibit growth of the PfMSP1-19 line. PcMSP1-19 specific rabbit antiserum and purified IgG specifically inhibited growth of the PcMSP1-19 line. The AMA1 MAb which recognizes a region of *P. falciparum *AMA1 domain 1 common to both the 3D7 and D10 parasite lines showed similar levels of growth inhibition of the PfMSP1-19 and PcMSP1-19 lines at a concentration of 0.5 mg/ml. The reproducibility of PfMSP1-19 specific rabbit antibodies in growth inhibition assays was confirmed in repeat experiments throughout the course of this study in both one cycle and two cycle assays (Figure 2).

### Optimisation of flow cytometry based growth assays

Prior studies using transgenic parasites to measure MSP1-19 specific inhibitory antibodies have been largely performed using microscopy-based assays, rather than by flow-cytometry, and there are limited data on conditions that influence growth-inhibition assay results or on assay reproducibility. Therefore, studies were performed to optimize flow cytometry-based growth inhibition assays. Different assay start parasitaemias were examined in order to determine the optimal conditions for measuring parasite growth inhibition. PfMSP1-19 parasite growth over one cycle in the presence of rabbit antisera or malaria exposed human samples showed a strong correlation between experiments using a starting parasitaemia of 0.5% and 1% (r = 0.95, n = 13, p < 0.0001). Two cycle assays set up at 0.05%, 0.1% and 0.3% parasitaemia showed a strong correlation between PfMSP1-19 growth for the 0.05% and 0.1% assays (r = 0.95, n = 13, p < 0.0001) and the 0.1% and 0.3% assays (r = 0.98, n = 8, p < 0.0001). These data suggest the start parasitaemia was unlikely to influence the sensitivity of parasite growth inhibition assays over the range of starting parasitaemia tested.

**Figure 3:**
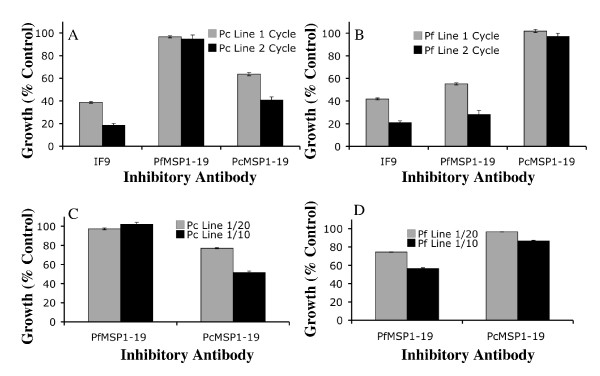
**Optimisation of growth inhibition assays**. Comparison of one and two cycle parasite growth inhibition assays using the 3D7 AMA1 (1F9) monoclonal antibody (0.5 mg/ml), PfMSP1-19 rabbit antiserum (1 in 10 dilution) and PcMSP1-19 rabbit antiserum (1 in 10 dilution) for **(A) **the PcMSP1-19 parasite line and **(B) **the PfMSP1-19 parasite line. Comparison of parasite growth inhibition by samples tested at either 1 in 10 or 1 in 20 dilution in a one cycle growth assay for **(C) **the PcMSP1-19 and **(D) **the PfMSP1-19 parasite lines. Growth is expressed as a percentage of controls (sera from non-exposed Australian residents). The mean of duplicate or multiple values is shown and error bars represent the range when tested in duplicate or standard error of the mean for 4 or more values as appropriate.

MSP1-19 specific rabbit antisera and the AMA1 MAb were compared in one cycle and two cycle growth inhibition assays to determine whether a second cycle of parasite growth would increase the sensitivity of the assay. Growth of the PcMSP1-19 parasite line in the presence of inhibitory antibodies showed that a second cycle increased antibody mediated growth inhibition for both the PcMSP1-19 and AMA1 antibodies but not the PfMSP1-19 antibodies (Figure 3A). Similarly, growth of the PfMSP1-19 line was considerably lower after two cycles in the presence of the PfMSP1-19 and AMA1 specific antibodies but not the PcMSP1-19 antibodies (Figure 3B). These data suggest that a second cycle of parasite growth increases growth inhibition due to antibodies compared to one cycle assays. Inhibition by MSP1-19 specific antibodies over two cycles of parasite replication was approximately double that observed in one cycle assays.

A number of published studies comparing growth of the PcMSP1-19 line and the PfMSP1-19 line have used a 1 in 20 volume of sample in one cycle microscopy based assays [36-39]. For the purposes of this study, an assay set up modified from Perrson *et al *[43] using a 1 in 10 sample dilution was compared to a 1 in 20 sample dilution in one cycle flow cytometry based assays with MSP1-19 specific rabbit antisera. Growth of the PcMSP1-19 transfected line when PcMSP1-19 antisera was added was higher for the 1 in 20 dilution assay than the 1 in 10 dilution assay (Figure 3C). Similarly PfMSP1-19 line growth in the presence of PfMSP1-19 antisera at a 1 in 20 dilution was higher than at a 1 in 10 dilution (Figure 3D). The specificity of the MSP1-19 rabbit antisera was confirmed by the absence of a dose-response inhibitory effect on the control MSP1-19 line for PfMSP1-19 or PcMSP1-19 antibodies (i.e. PfMSP1-19 antibodies did not substantially inhibit the PcMSP1-19 line, or vice-versa). Similarly, median growth of the PfMSP1-19 transfected parasite line in a 1 in 10 dilution experiment for a panel of malaria exposed non-dialysed adult samples (n = 15) was lower (growth 59.2%) compared to the median growth for 1 in 20 dilution experiments (growth 85.6%) relative to control in one cycle experiments indicating an inhibitory dose response effect of human samples on parasite growth.

### Transfection of MSP1-19 chimeric lines with green fluorescent protein (GFP)

**Figure 4:**
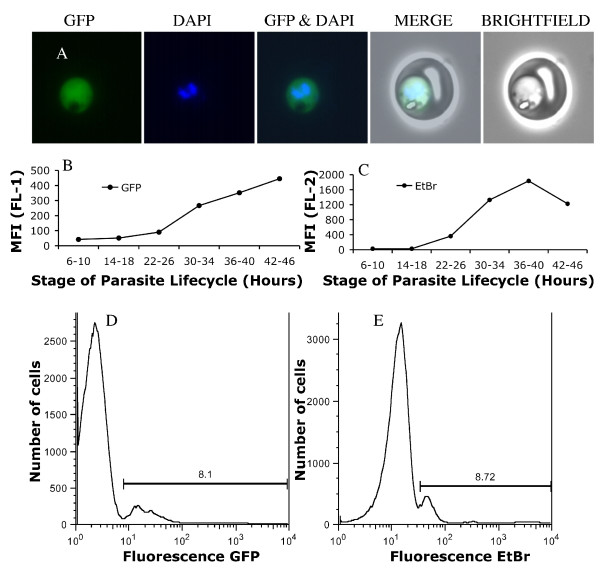
**GFP fluorescent *P. falciparum *and changes in fluorescence over the course of the parasite asexual lifecycle**. **(A) **Trophozoite stage PfM3' parasite transfected with the pHGBrHrBl-1/2 plasmid expressing GFP. Selected images show GFP fluorescence, DAPI staining of the nucleus, and bright field image showing the infected erythrocyte. Merged images demonstrate GFP fluorescence appears largely restricted to the parasite cytosol. **(B) **Changes in GFP mean fluorescence intensity of tightly synchronized PfPHG parasites followed through 48 hours of the parasite lifecycle by flow cytometry (detected in channel Fl-1). **(C) **Changes in EtBr mean fluorescent intensity (detected in channel FL-2) of the same parasites stained with 10 μg/ml EtBr followed through 48 hours of the parasite lifecycle. Detection of early ring stage parasites by flow cytometry using **(D) **GFP fluorescence or **(E) **EtBr fluorescence highlighting the poor separation commonly seen between EtBr stained rings and uninfected RBCs.

The PfM3' and PcMEGF MSP1-19 lines developed by O'Donnell *et al *[39,51] were transfected with a plasmid expressing GFP under Blasticidin HCl drug selection (transfection of PfM3' leading to PfPHG, transfection of PcMEGF leading to PcPHG). Fluorescence microscopy indicated that GFP fluorescence was not targeted to specific organelles but instead coloured the whole parasite cytoplasm (Figure 4A). GFP fluorescent parasites were detectable in all asexual stages of the life cycle.

The fluorescence profile of both EtBr stained and unstained PfPHG GFP expressing parasites was examined during the course of the asexual parasite lifecycle. GFP fluorescence profiles for early ring stage parasites (2, 6-10, 14-18 hours post invasion) separated clearly from the uninfected RBC population (Figure 4B,D). This allowed the parasitaemia of GFP fluorescent parasites to be measured by flow cytometry at any stage of the parasite life cycle, including early rings. As the parasites matured, the mean fluorescence intensity (MFI) of GFP positive parasites increased. There was a consistent increase in MFI associated with time post invasion (Figure 4B). GFP fluorescence was not influenced by whether or not the parasites had been stained with EtBr. EtBr stained parasite fluorescence peaked around 36-40 hours post invasion, with a noticeable increase in mean fluorescence intensity from approximately 22-26 hours to 36-40 hours post invasion (Figure 4C). Early ring stage parasites (0-12 hours post invasion) stained with EtBr generally separated poorly from the RBC population (Figure 4E). Although the vector was designed to transfect the D10 PcMEGF and PfM3' lines, the same vector was successfully used to transfect other parasite lines, CS2 and E8B; fluorescence was confirmed by flow cytometry and immunofluorescence microscopy. Other vectors could be generated for specific applications.

### Validation of GFP fluorescent parasites in growth assays using inhibitory antibodies and anti-malarial drugs

There was a strong correlation between GFP fluorescence and EtBr staining for measurements of PfPHG parasite growth in two cycle assays (r = 0.97, n = 57, p < 0.0001) in the presence of inhibitory and non-inhibitory samples. Growth of the PfPHG GFP expressing line also correlated strongly with growth of the PfM3' parent line in inhibitory and non-inhibitory samples for two cycle assays (r = 0.88, n = 27, p < 0.0001).

**Figure 5:**
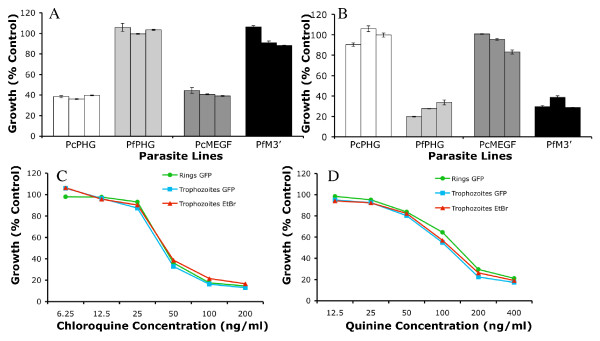
**Measurement of growth inhibitory activity of antibodies and anti-malarial drugs using GFP fluorescent parasites**. Comparison between the GFP transfected PcPHG and PfPHG lines and the parental parasites (PcMEGF and PfM3', respectively) of growth-inhibitory activity of **(A) **PcMSP1-19 specific rabbit antisera and **(B) **PfMSP1-19 specific rabbit antisera. Results represent data from three independent two cycle growth assays with samples tested at 1 in 10 dilution. Growth is expressed as a percentage of controls (sera from non-exposed Australian residents) and values represent means with SEM. Inhibition of the PfPHG parasite line by **(C) **chloroquine and **(D) **quinine in a one cycle growth assay. Parasitaemia was measured by flow cytometry using ring stage GFP fluorescence, late trophozoite stage GFP fluorescence or late trophozoite stage EtBr fluorescence. Growth is expressed as a percentage of PBS control.

Inhibition of parasite growth by antibodies for the PcPHG and PfPHG fluorescent lines was compared to inhibition of the PcMEGF and the PfM3' parent lines in two cycle assays. Growth of the PcPHG GFP expressing line was found to be the same as the PcMEGF non-GFP parent line in the presence of *P. chabaudi *MSP1-19 specific antiserum, *P. falciparum *MSP1-19 specific antiserum (Figure 5A) and AMA1 MAb. Similarly, growth of the PfPHG GFP expressing line and the PfM3' non-GFP parent line was found to be the same in the presence of *P. chabaudi *MSP1-19 specific antiserum, *P. falciparum *MSP1-19 specific antiserum (Figure 5B) and AMA1 MAb, as expected. The level of inhibition between the parent and GFP transfected lines in the presence of MSP1-19 specific rabbit antisera was reproducible over multiple assays.

The utility of GFP-fluorescent lines in growth assays was also evaluated with anti-malarial drugs. The growth of PfPHG parasites in the presence of the anti-malarial drugs chloroquine (Figure 5C) and quinine (Figure 5D) showed a strong correlation between measurements made at ring stage (based on GFP fluorescence) and mature trophozoite stage detected by GFP fluorescence or EtBr fluorescence. The correlation between parasitaemia measured by GFP and EtBr in the presence of chloroquine and quinine indicated that the two methods were equally sensitive. There was no evidence that either method was significantly overstating parasitaemia due to increased background reading.

Taken together, these data suggest that expression of GFP does not alter parasite susceptibility to inhibitory antibodies or anti-malarial drugs. GFP fluorescent parasites are potentially a useful tool for measurement of parasite growth inhibition in *in vitro *assays.

### Measurement of parasitaemia by microscopy versus flow cytometry

**Figure 6:**
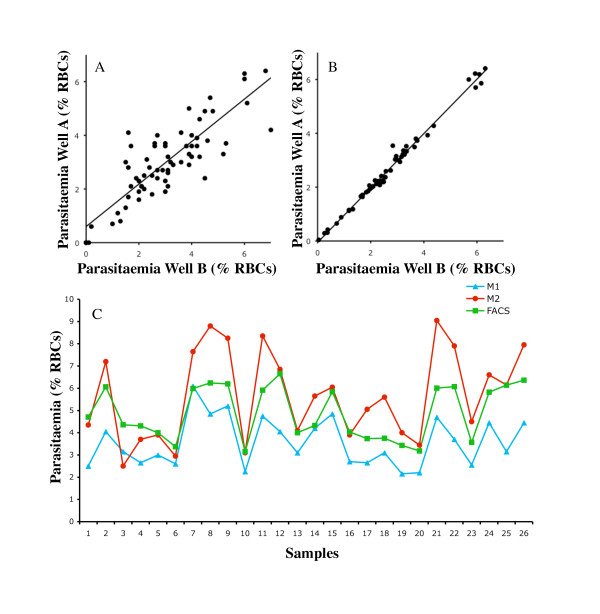
**Measurement of parasite growth inhibition by microscopy and flow cytometry**. Correlation between duplicate wells of parasitaemia counts from a growth inhibition assay using **(A) **ring stage microscopy (n = 70) or **(B) **flow cytometry (n = 55). The higher number of samples available for ring stage microscopy reflects the availability of counts from a second microscopist. The PfMSP1-19 line was used in this comparison and tested for inhibition by rabbit anti-MSP1-19 antibodies and a selection of samples from malaria-exposed and non-exposed donors. **(C) **Mean parasitaemia counts for duplicate wells determined by ring stage microscopy counts for microscopist one (M1) and microscopist two (M2) compared to parasitaemia measured by flow cytometry (trophozoite stage parasites) using the PcMSP1-19 and PfMSP1-19 lines. Samples used were serum from malaria exposed individuals and unexposed controls numbered 1-26 for ease of comparison.

Further studies were performed to evaluate flow-cytometry-based measurements of parasitaemia compared to microscopy. In total, 55 samples, including inhibitory antibodies and serum samples from malaria-exposed and non-exposed donors were compared between microscopy and flow cytometry based measurements of parasite growth for duplicate wells across three experiments. Counts for 15 samples by a second microscopist were available for comparison. There was noticeable variation in measurements by microscopy between duplicate wells for both the PcMSP1-19 line (correlation between wells r = 0.76, n = 70, p < 0.0001, mean difference in parasitaemia between duplicate wells 0.8%, range 0% to 5.5%) and the PfMSP1-19 line (r = 0.80, n = 70, p < 0.0001, mean difference in parasitaemia between duplicate wells 0.7%, range 0% to 2.8%, Figure 6A). In contrast, for flow cytometry based assays, there was a very strong correlation between measurements of parasitaemia for duplicate wells for both the PcMSP1-19 (r = 0.99, n = 55, p < 0.0001, mean difference in parasitaemia between duplicate wells 0.1%, range 0% to 0.3%) and PfMSP1-19 (r = 0.98, n = 55, p < 0.0001, mean difference in parasitaemia between duplicate wells 0.1%, range 0% to 0.7%, Figure 6B) lines.

The reproducibility of parasitaemia measurements was examined for a single experiment where counts for duplicate wells were available for two microscopists (M1 and M2) and a flow cytometry based assay. Measurements of parasitaemia between duplicate wells were compared for all samples and control treatments (n = 13) in the experiment for both the PcMEGF and PfM3' parasite lines. The mean percentage difference in parasitaemia counts between duplicates wells for all treatments and both lines in the flow cytometry based assay was 0.2% (range 0% to 0.7%). The mean percentage difference between duplicate wells across all serum treatments for M1 was 0.6% (range 0% to 1.6%). For M2 the mean percentage difference between duplicate wells across all serum treatments was 1.5% (range 0% to 5.5%).

Comparison between microscopist M1, microscopist M2 and flow cytometry based counts of parasitaemia (mean of duplicate wells) suggested that the counts for M1 (mean of all samples 3.6%) tended to underestimate parasitaemia relative to flow cytometry counts (mean 4.9%), while the counts of M2 (mean 5.7%) tended to overestimate parasitaemia relative to flow cytometry (Figure 6C). The comparison also highlights where parasitaemia counts from both microscopists differed widely to that of the flow cytometry parasitaemia counts for individual samples.

## Discussion

In this study, flow cytometry-based growth inhibition assays [43] were further evaluated and optimized and GFP-fluorescent lines were developed and validated for use in measuring growth-inhibitory antibodies and the activity of anti-malarial agents. Inhibition of parasite growth in the presence of MSP1-19 specific rabbit antisera [39], AMA1 MAb [49,50] and human samples from malaria-exposed individuals was higher in two cycle assays than in one cycle assays, as previously reported [43]. Similarly, MSP1-19 specific rabbit antisera and human samples were more inhibitory at a 1 in 10 dilution than a 1 in 20 dilution. Assay starting parasitaemia did not appear to affect the level of parasite growth inhibition for inhibitory samples. There was a strong correlation between duplicate wells and repeat experiments for both one cycle and two cycle flow cytometry based assays as reported previously [43,48]. Importantly, the level of inhibition for all three inhibitory antibodies was found to be highly reproducible throughout the course of the study for both one cycle and two cycle assays.

GFP fluorescent PcMSP1-19 and PfMSP1-19 parasites were developed to reduce handling and improve sensitivity in flow cytometry based growth assays; parasites were transfected to express GFP under control of the HSP-86 promoter on both the PcMEGF (PcPHG) and PfM3' (PfPHG) parent line backgrounds and parasites were cloned for subsequent use. Parasites expressing cytoplasmic GFP were easily detectable by flow cytometry and measurements of parasitaemia correlated strongly between GFP fluorescent parasites and parasites detected using the established method of staining with ethidium bromide.

There are several advantages of using GFP-fluorescence compared to nucleic acid dyes to detect parasitaemia. The use of the GFP fluorescent parasites removed the need to use a nucleic acid stain for parasite detection by flow cytometry, thereby reducing handling, and gave better resolution between ring stage parasites and uninfected RBCs by flow cytometry compared to EtBr stained rings. Furthermore, fluorescent parasites can be sorted using flow cytometry and used in down-stream applications that require live viable parasites, which can be problematic with nucleic acid dyes [59,60]. Detection of GFP fluorescent parasites was found to be as reliable as detection using EtBr stain in flow cytometry based growth assays, with strong correlations between measured parasitaemia for both one cycle and two cycle growth assays. Growth of GFP transfected and parental parasites was found to be comparable in the presence of MSP1-19 specific inhibitory rabbit antisera, inhibitory AMA1 MAb and malaria exposed human samples. This suggests that parasites expressing GFP are a valid and reliable alternative to the use of a nucleic acid stain such as EtBr for counting parasitaemia by flow cytometry in *in vitr*o assays. These studies demonstrate that GFP-fluorescent parasites could be used in assays to measure acquired immunity or vaccine-induced responses in the development and testing of blood-stage vaccines, or for screening and evaluation of compounds with anti-malarial activity.

Finally, the measurement of parasitaemia by flow cytometry was found to be more accurate and reproducible than microscopy. Reproducibility of parasitaemia counts between duplicate wells was much higher for flow cytometry than microscopy. Differences in parasitaemia counts between duplicate wells of the same sample were as high as 98% by microscopy, whereas parasitaemia counts by flow cytometry were very similar for duplicate wells of the same sample, suggesting that much of the variation between duplicate wells in microscopy is caused by observer error. There was also substantial variability in results between microscopists.

## Conclusions

The development and optimisation of flow cytometry based growth inhibition assays using parasite lines transfected to express GFP resulted in a highly reproducible assay that was sensitive and suitable for measuring the inhibitory activity of antibodies and anti-malarial agents. Results were highly reproducible between experiments, between duplicate samples and superior to microscopy.

## Competing interests

The authors declare that they have no competing interests.

## Authors' contributions

DWW - study design, analysis and interpretation of results, performed experiments, wrote the manuscript with JGB; BSC - study design, interpretation of results and contributed to writing the manuscript; JGB - study design, analysis and interpretation of results, wrote the manuscript with DWW. All authors read and approved the final manuscript.
